# Study of Well Waters from High-Level Natural Radiation Areas in Northern Vietnam

**DOI:** 10.3390/ijerph18020469

**Published:** 2021-01-08

**Authors:** Van-Hao Duong, Thanh-Duong Nguyen, Miklos Hegedus, Erika Kocsis, Tibor Kovacs

**Affiliations:** 1 Geophysics Department, Hanoi University of Mining and Geology, No 18, Vien Street, Bac Tu Liem District, Hanoi 100 000, Vietnam; duongvanhao@humg.edu.vn (V.-H.D.); nguyenthanhduong@humg.edu.vn (T.-D.N.); 2 Institute of Radiochemistry and Radioecology, University of Pannonia, H-8200 Veszprem, Hungary; hegedusm@almos.uni-pannon.hu (M.H.); kocsiserika@almos.uni-pannon.hu (E.K.)

**Keywords:** ^226^Ra, ^228^Ra, ^238^U, well water, radiological hazards, REE and uranium mines, northern Vietnam

## Abstract

The determination of natural radionuclide concentrations plays an important role for assuring public health and in the estimation of the radiological hazards. This is especially true for high level radiation areas. In this study, ^226^Ra, ^228^Ra and ^238^U concentrations were measured in well waters surrounding eight of the high-level natural radiation areas in northern Vietnam. The ^226^Ra, ^228^Ra and ^238^U activity concentrations vary from <1.2 × 10^−3^–2.7 (0.46), <2.6 × 10^−3^–0.43 (0.07) and <38 × 10^−3^–5.32 Bq/L (0.50 of median), respectively. ^226^Ra and ^238^U isotopes in most areas are in equilibrium, except for the DT-Thai Nguyen area. The calculated radiological hazard indices are generally higher than WHO (World Health Organization) recommendations. Average annual effective dose and excess lifetime cancer risk values due to drinking well water range from to 130 to 540 μSv/year and 7.4 × 10^−6^ to 3.1 × 10^−5^, respectively.

## 1. Introduction

Human beings are always exposed to a wide range of natural radionuclides [1]. Natural radionuclides can be present in the whole environment, including soil, water, air, food and even our bodies. Radionuclides in soil, air and water come from different sources, such as the weathering of the earth’s crust, mining activities or fertilizer materials [2,3,4,5,6,7]. The radionuclides in water can enter the food chain, if the water is used for drinking or irrigation purposes. Determination of natural radionuclide concentrations in all the environments plays an important role for public health, because it can be used to assess the population’s exposure to radiation and estimate the radiological hazard.

Investigations on natural radiation have received particular attention throughout the world in the last decade, which led to extensive studies in many countries, especially in or surrounding the high-level natural radiation areas. Studies regarding the natural radioactivity in water from different sources were widely conducted [7,8,9,10,11,12,13,14,15].

Among natural radionuclides, uranium leaches out from the bedrock and is present in water (surface and underground water) in various dissolved and suspended particulate forms. Other sources can be from the dry or wet deposition of aerosol from air. ^228^Ra originates from the ^232^Th series, and in contrast to the typically not very soluble of Th element, ^228^Ra can be partially mobilized in natural waters, giving information on geochemical conditions and enabling contributions to the potential public exposure. ^226^Ra is a long-lived daughter of the ^238^U decay series, and it is also found in the water in trace quantities. The concentrations of ^238^U, ^228^Ra and ^226^Ra in the water depend on the lithology, geomorphology and other geological conditions [16]. Thus, the concentration of these radionuclides varies from one site to another. The study concerning ^226^Ra, ^228^Ra and ^238^U concentrations in drinking water allows understanding their distribution and evaluating their impact on human health.

In Northern Vietnam, there are several mines, which contain higher than average concentrations of radioactive elements such as the rare earth mines in NX (Lai Chau), DP (Lai Chau), MH (Lao Cai) and YP (Yen Bai); there is also a polymetallic mine (also containing high uranium concentration) in DT (Thai Nguyen); finally, there is uranium ore in BY (Son La), TS (Phu Tho) and NB (Cao Bang). These mines were recently reported to have a high radioactive background by unpublished data from the Geological Division for Radioactive and Rare Minerals, Hanoi, Vietnam. This presents a possible public health concern. Therefore, in this study, the natural radionuclide concentrations in well water (^226^Ra, ^228^Ra ^238^U) in the area surrounding these mines are investigated. Based on the activity concentrations, the radiological health hazards are also evaluated.

## 2. Materials and Methods

### 2.1. Study Areas

**Figure 1:**
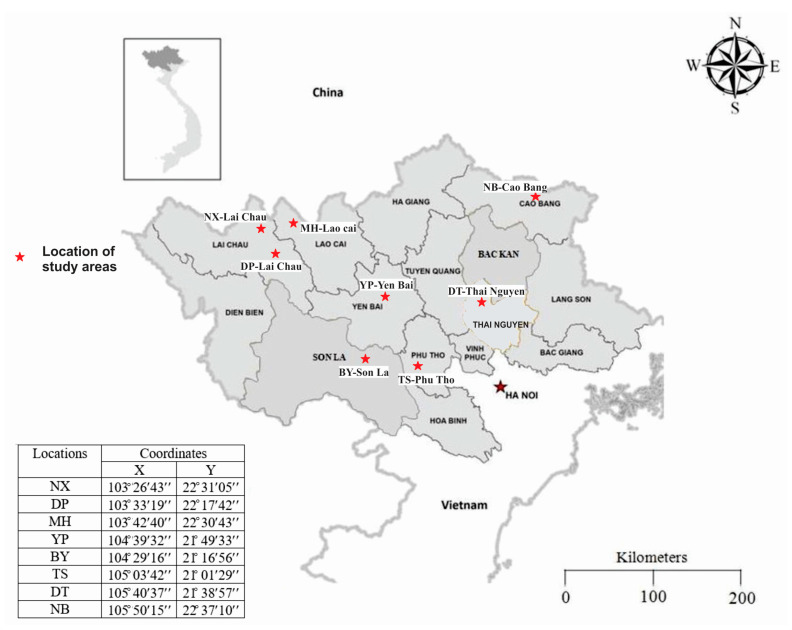
Location of the study areas (map was modified from Hung et al., 2016) [18].

The eight areas in Northern Vietnam, including NX-Lai Chau, DP-Lai Chau, MH-Lao Cai, BY-Son La, TS-Phu Tho, YP-Yen Bai, DT-Thai Nguyen and NB-Cao Bang were selected for this study. The location of these areas is presented in Figure 1. The NX mine is one of the largest rare earth element (REE) mines in Vietnam, with probable reserves of about 7.7 million tons. DP mine ranks the second, with probable reserves of about 3.7 million tons and is followed by MH with approximately 400,000 tons and YP with about 5000 tons [17]. BY (Son La), TS (Phu Tho) and NB (Cao Bang) have uranium ore deposits, while DT (Thai Nguyen) is the largest polymetallic mine in Vietnam.

### 2.2. Sample Collection and Preparation

In each study location, 20 water samples were collected from local wells during 2018–2019. These wells were dug manually in the soil to the depth of about 5 to 10 m, and these wells provide drinking water for the local population. A total of 160 water samples with 50 L for each sample were collected for this study. Each water sample was stored in a big, 50 L plastic container. Each water sample was co-precipitated as Ba(Ra)SO_4_ for radium isotopes, then the uranium isotopes were subsequently precipitated as (NH_4_)_2_U_2_O_7_ together with MnO_2_ [15,19,20]. The solid precipitate was then filtered. Together with study samples, a blank sample was prepared using distilled water in order to determine the background. The obtained precipitated sample was dried and milled to powder, then they were pressed into cylindrical plastic containers, weighted and finally hermetically sealed. The samples were stored for 4 half-lives in order to reach the secular equilibrium (16 days for ^226^Ra after sealing, and approximately 100 days for ^238^U after precipitation).

### 2.3. Methods

#### 2.3.1. Measurements of Activity Concentration of ^238^U, ^228^Ra and ^226^Ra in Water

After the samples reached equilibrium, activity concentration measurements were performed using a high-resolution detector HPGe with a low background made by Ortec™. The analysis was performed using Gamma Vision software. The detector’s energy resolution was 1.9 keV at the 1.33 MeV ^60^Co gamma-ray peak. To reduce the effects of background radiation at the laboratory, the detector was shielded by a 10-cm thick old-lead cylinder with a 1 mm cadmium and 1 mm copper inner lining. The samples were counted for two days to minimize the statistical counting error and activity calculation and calibration were carried out based on standard reference materials (IAEA-375). The level of background radiation present in the laboratory and introduced by the chemical process was determined using the blank sample.

The activity concentration of each sample was determined based on its respective gamma lines. The gamma lines of 609.3 keV, 1120.3 keV and 1764.5 keV of ^214^Bi were used to determine the activity concentration of ^226^Ra, the 911.1 keV line of ^228^Ac was used for ^228^Ra while the 1001 keV line of ^234m^Pa was used for ^238^U (which was verified by ^235^U measurement using the 186 keV line). The lowest limit detection were 0.0012, 0.0026 and 0.038 Bq/L for ^226^Ra, ^228^Ra and ^238^U, respectively (the values were used for a studied sample volume of 50 L).

The activity concentrations of ^226^Ra, ^228^Ra and ^238^U are calculated based on the following Equation (1) [7]:

(1) $$A_{\mathrm{sp}}=\frac{N_{\mathrm{sp}}M_{\mathrm{st}}A_{\mathrm{st}}C_{i}C_{\mathrm{di}}}{N_{\mathrm{st}}M_{\mathrm{sp}}}$$

where: A_sp_ and A_st_ is activity concentration of studied and standard samples; N_sp_, M_sp_ and N_st,_ M_st_ are the net measured intensity and mass of the sample and standard sample, respectively; C_i_ is the correction factor for the differences between the densities of the samples and the standard sample for the i isotope; and C_di_ is the correction fraction for the precipitation efficiency for the i isotope.

#### 2.3.2. Evaluation of Radiological Hazard Indices

- Annual effective dose (AED)

The annual effective dose (AED) due to the ingestion of the drinking well water was estimated to assess the radiological hazards for the local population by using Equation (2) [21]:

AED (μSv/year) = A (Bq/L) × Cw (L/year) × DCF (μSv/Bq)(2)

where AED is the annual effective dose due to ingestion of radionuclides; A is the activity concentration of radionuclides; Cw is the annual water consumption for a person (730 L/year for adults) [22]. DCF is the ingestion dose conversion factor for the corresponding radionuclides (0.28, 0.69 and 0.045 μSv/Bq for ^226^Ra, ^228^Ra and ^238^U, respectively) [21,23]. We all know that there are some other isotopes, like ^210^Po, which can contribute to a higher annual effective dose caused by drinking well waters, but in this study we only used the ^226^Ra, ^228^Ra and ^238^U values to calculate the AED.

- Excess lifetime cancer risk (ELCR)

Based on the values of AED, excess lifetime cancer risks (ELCR) were calculated using the following Equation (3) [24]:

ELCR = AED × Life Expectancy (LE) × Risk factor (RF)(3)

where LE is life expectancy of Vietnamese people in North Vietnam and mountainous areas (71 years) (https://www.gso.gov.vn/default_en.aspx?tabid=774); RF the risk factor associated with radiation, which is equal to 0.057 Sv^−1^ [24].

## 3. Results and Discussion

### 3.1. Activity Concentration

The range and average values of activity concentration of ^226^Ra, ^228^Ra and ^238^U measured in the well water samples are given in Table 1. It can be seen that the activity concentration of ^226^Ra, ^228^Ra and ^238^U ranges from <0.0012–2.7, <0.0026–0.43 and <0.038–5.32 Bq/L, respectively. The highest concentrations of all three isotopes are found in DT-Thai Nguyen. This table shows only a slight difference in concentration between ^226^Ra, ^228^Ra and ^238^U in most cases, except for the DT-Thai Nguyen sampling site. ^226^Ra, ^228^Ra and ^238^U ratios near unity indicate recent contact with uranium bearing not yet weathered minerals [25]. The concentrations of ^226^Ra, ^228^Ra and ^238^U are less than 1 Bq/L in most areas, except for DT-Thai Nguyen (Table 1). In the case of DT-Thai Nguyen, the concentrations of ^226^Ra, ^228^Ra and ^238^U are comparatively high and are in the ranges of 0.36–2.70, 0.05–0.43 and 0.33–5.32 Bq/L, respectively. There, the ^226^Ra concentration can reach levels multiple times higher than the WHO guideline (1 Bq/L) [26]. The high concentrations of ^226^Ra and ^238^U in DT-Thai Nguyen can be attributed to the polymetallic mine (which contains high uranium concentration) in this area. There are some activities, such as exploitation and the process of ore sorting going on, which can influence activity concentrations. It should be noted that the water samples in this study were taken from wells with depth of less than 10 m. These type of wells depend on rainfall and surface water as their source of water. Accordingly, they are easily contaminated by surface water and various human activities. Thus, the human activities in the polymetallic mine can lead to a relatively high concentration of ^226^Ra, ^228^Ra and ^238^U in well water.

Table 2 compares the ^226^Ra, ^228^Ra and ^238^U concentrations in the well water samples in this study with that of different water sources in different countries. The concentrations of ^226^Ra, ^228^Ra and ^238^U in well water in the areas observed in this study are significantly higher than those in Hoa Binh, Vietnam. In addition, the observed concentrations are higher than those in reported for many other countries [8,9,10,11,12,16], whereas they are lower than some values reported for tube wells in India. The concentrations observed in well water significantly depend on the type of aquifer rock as well as the chemical and physical characteristics of water [27], thus such differences can be expected. The concentration of studied radionuclides observed in well water in this study is within the worldwide range [28].

Regarding the concentration ratio of ^226^Ra/^238^U in well water samples, as shown in Table 1, the average value ranges from 0.57 (DT-Thai Nguyen) to 1.09 (BY-Son La). The data presented in Table 1 also shows that on average there is near equilibrium between ^226^Ra and ^238^U, except for DT-Thai Nguyen. Kumar et al. (2016) reported that the concentration of ^226^Ra/^238^U in groundwater in southwestern Punjab in India was varied from 0.08 to 0.22 [29]. In groundwater in Finland, Asikainen (1981) also showed that the ratio of ^226^Ra/^238^U ranged from 0.05 to 1. By contrast, other previous studies reported the enrichment of ^226^Ra in groundwater [30]. For examples, Gascoyne (1989) indicated that the ^226^Ra/^238^U ratios in Canadian groundwater varied from 0.026 to 5300; this ratio in Konnngara Australian groundwater was from 0.02 to 89 [31]. Recently, the research results of Almasoud et al. (2020) indicated that the ratios of ^226^Ra/^238^U in groundwater samples in Saudi Arabia ranged from 1.25 to 20.4 [32]. The issue is further complicated by the effects of the recoil from the emission of an alpha particle, which can increase the mobility of the daughter nuclide due to the Szilárd–Chalmer effect. On the other hand, the ^234^Th or ^234^U can be fixed to more weathering resistant mineral phases, resulting in relatively more ^238^U dissolving into groundwater [31]. The depletion of _234_U in groundwater can also be observed based on the relative abundances of U under various geochemical conditions [30]

**Figure 2:**
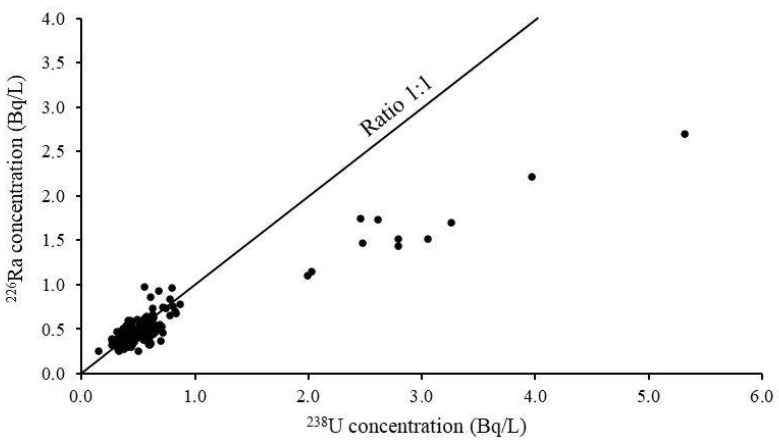
Relationship between ^238^U and ^226^Ra concentrations.

The relationship between activity concentrations of ^238^U and ^226^Ra in well water samples in this study is shown in Figure 2. A significant positive correlation was found between the two radionuclides with a Pearson correlation coefficient, 0.9402 and a *p* value < 0.00001 for the overall dataset, due to the influence of the higher values observed at DT-Thai Nguyen. The high value of correlation between ^238^U and ^226^Ra shows that these radionuclides have leached from the similar host rock [16]. Excluding DT-Thai Nguyen, there is moderate positive correlation with a Pearson correlation coefficient of 0.6326, and a *p* value < 0.00001. Similarly, a strong positive correlation was observed both between ^238^U and ^228^Ra (Pearson correlation coefficient: 0.8411, with a *p* value < 0.00001) and ^226^Ra and ^228^Ra (Pearson correlation coefficient: 0.7834, with a *p* value < 0.00001) for the overall dataset, however the effect of the higher values at DT-Thai Nguyen improving the correlation are observable here as well.

### 3.2. Radiological Hazards

The calculated radiation hazard indices based on the average activity concentrations for some drinking well water in northern Vietnam are listed in Table 3. As shown in this table, the annual effective dose (AED) for ^226^Ra is significantly higher than that for ^238^U, while ^228^Ra is in the middle despite having a higher dose conversion coefficient due to the comparatively low activity concentrations. The average total annual effective dose for adults due to the consumption of water ranges from 130 to 540 μSv/year with the mean value of 240 μSv/year. The average excess life cancer risk (ELCR) due to drinking the investigated well water is from 7.4 × 10^−6^ to 3.1 × 10^−5^ (7 to 31 cases per 1 million people) with the average of 1.4 × 10^−5^ (14 cases per 1 million people). Specific wells can have higher values; the overall maximum activity concentrations were observed in a well in YP-Yen Bai translating to a total annual effective dose of 540 μSv/y for adults and an ELCR of 7.0 × 10^−5^ (70 cases per 1 million people). As reported by the WHO (2017), the reference values for AED and ELCR due to drinking water are 100 μSv/year and 1.0 × 10^−5^, respectively. It can be seen that the results of AED and ELCR due to consumption of well water in this study are higher on average for each area from the observed radionuclides alone than the values suggested by the WHO (2017), with the exception of ELCR for YP-Yen Bai. This indicates that there is a need for defining local policy regarding the wells in high-level natural radiation areas, northern Vietnam (in the observed areas), especially DT-Thai Nguyen.

## 4. Conclusions

The concentrations of ^226^Ra, ^228^Ra and ^238^U in well waters in different locations surrounding the high-level radiation areas in northern Vietnam were extensively measured and evaluated. The research results show that the concentrations of ^226^Ra, ^228^Ra and ^238^U in well water samples in the observed mining areas of northern Vietnam are comparatively higher than those reported for other areas of Vietnam and other countries. The highest concentrations of ^226^Ra, ^228^Ra and ^238^U are observed in DT-Thai Nguyen. The research also shows that the concentration of ^226^Ra and ^238^U for most locations on average are around equilibrium, except for DT-Thai Nguyen. Regarding the radiological hazards assessment, the calculated results of AED and ELCR due to the consumption of well water are often higher, and for DT-Thai Nguyen multiple times higher, than the WHO reference values. The results generated from this study provide important baseline data for the impact assessment of the mining activities in the region in the future.

## Figures and Tables

**Table 1:** Concentration of natural radionuclides in well water samples in North, Vietnam.

Locations	Type of Mine	Value		Activity Concentration (Bq/L)		^226^Ra/^238^U
			^226^Ra	^228^Ra	^238^U	
NX-Lai Chau	REE mine	Range (SD)	0.26–0.65 (0.09)	0.04–0.10 (0.01)	0.15–0.72 (0.15)	0.64–1.73
		Average	0.44	0.06	0.50	0.95
DP-Lai Chau	REE mine	Range (SD)	0.35–0.59 (0.08)	0.05–0.15 (0.03)	0.31–0.71 (0.10)	0.60–1.19
		Average	0.47	0.11	0.54	0.90
MH-Lao Cai	REE mine	Range (SD)	0.30–0.78 (0.16)	<0.0026–0.11 (0.02)	0.31–0.87 (0.18)	0.69–1.52
		Average	0.52	0.07 *	0.56	0.96
YP-Yen Bai	REE mine	Range (SD)	<0.0012–0.54 (0.07)	<0.0026–0.12 (0.02)	<0.038–0.70 (0.12)	**
		Average	0.23 *	0.08 *	0.31 *	**
BY-Son La	Uranium mine	Range (SD)	0.25–0.74 (0.11)	<0.0026–0.09 (0.02)	0.27–0.63 (0.08)	0.76–1.44
		Average	0.45	0.06 *	0.41	1.08
TS-Phu Tho	Uranium mine	Range (SD)	0.25–0.97 (0.19)	0.05–0.10 (0.02)	0.27–0.69 (0.11)	0.50–1.76
		Average	0.48	0.07	0.48	1.01
DT-Thai Nguyen	Uranium mine	Range (SD)	0.36–2.70 (0.69)	0.05–0.43 (0.11)	0.33–5.32 (1.46)	0.50–1.42
		Average	1.15	0.18	2.06	0.79
NB-Cao Bang	Uranium mine	Range (SD)	0.32–0.97 (0.18)	<0.0026–0.13 (0.02)	0.34–0.80 (0.12)	0.53–1.43
		Average	0.53	0.07	0.55	0.97
Overall range	Minimum		<0.0012	<0.0026	<0.038	0.50
	Maximum		2.7	0.43	5.32	1.76

* during averaging values under the detection limit were taken as the detection limit to give a conservative estimate. ** uncalculable values were left out of the ratio calculation.

**Table 2:** ^226^Ra and ^238^U concentrations in water samples in different areas.

Countries	Samples	Activity concentration (Bq/L)			References
		^226^Ra	^228^Ra	^238^U	
Northern Vietnam	Well water	<0.0012–2.7	<0.0026–0.43	<0.038–5.32	This study
Hoa Binh, Vietnam	Groundwater	0.005–0.029	≤0.020	≤0.0005–0.009	[15]
Italy	Drinking water	0.0050–0.0608	0.00010–0.0257	0.000206–0.103	[12]
Turkey	Drinking water	<0.027–2.431	<0.036–0.270	-	[9]
Jordan	Tap water	0.096	0.170	0.033	[8]
Erbil, Iraq	Surface water	0.274–1.03	0.00676–0.244 *	0.274–1.03 *	[11]
Gogi, India	Tube well	0.0195–10.5	-	0.0123–33.2	[16]
	Open well	0.0366–0.0571	-	0.114–0.160	
Ghana	Groundwater	0.09–0.18	0.22–0.99 *	0.09–0.18 *	[10]
	Surface water	0.08–0.17	0.18–0.74 *	0.08–0.17 *	
World range	Drinking water	0.0002–45	0.0001–7.7	0.000028–150	[28]

* Equilibrium was assumed by the original authors.

**Table 3:** Radiation hazard indices for well water samples in northern Vietnam.

Locations	Type of Mine	AED (μSv/Year)				ELCR
		^226^Ra	^228^Ra	^238^U	Total	
NX-Lai Chau	REE mine	120	40	20	190	1.1 × 10^−5^
DP-Lai Chau	REE mine	130	80	20	240	1.3 × 10^−5^
MH-Lao Cai	REE mine	150	50	30	220	1.3 × 10^−5^
YP-Yen Bai	REE mine	60	50	10	130	7.4 × 10^−6^
BY-Son La	U mine	130	40	20	180	1.0 × 10^−5^
TS-Phu Tho	U mine	140	50	20	210	1.2 × 10^−5^
DT-Thai Nguyen	U mine	320	120	90	540	3.1 × 10^−5^
NB-Cao Bang	U mine	150	50	20	220	1.3 × 10^−5^
Average		150	60	30	240	1.4 × 10^−5^

## Data Availability

The data presented in this study are available on request from the corresponding author.
